# Factors Associated with Disordered Eating Behaviors among Triathletes

**DOI:** 10.51894/001c.5958

**Published:** 2017-08-24

**Authors:** Samantha Fugate Kennedy, Alyse Folino Ley, Brianna Nicole Fugate, Alexander Hayek

**Affiliations:** 1 Michigan State University Department of Psychiatry, Resident, East Lansing, MI; 2 Michigan State University Department of Psychiatry, Residency Director, East Lansing, MI; 3 Eaton Regional Education Service Agency School Psychologist, Charlotte, MI; 4 Michigan State University College of Osteopathic Medicine, Osteopathic Medical Student, East Lansing, MI

**Keywords:** athletes, bulimia nervosa, anorexia nervosa, disordered eating

## Abstract

**CONTEXT:**

Although there is little data currently available concerning the primary factors associated with disordered eating behaviors among triathletes, these athletes may be at greater risk. Sports medicine professionals are in a unique position to identify athletes with disordered eating risks.

**METHODS:**

The purpose of this cross-sectional survey study was to identify the prevalence of disordered eating attitudes and behaviors among a national convenience sample of triathletes. A secondary purpose was to identify “triathlon-specific factors” and “competitive athlete-factors” potentially associated with disordered eating behaviors. The authors hypothesized that certain triathlon-specific factors and competitive athlete factors would be associated with increased rates of self-reported disordered eating behaviors.

**RESULTS:**

In a respondent sample of 1,033 adults, multiple competitive athlete factors were associated with a higher risk for disordered eating, whereas triathlon-specific factors were not.

**CONCLUSIONS:**

In this study sample, disordered eating was not specifically associated with triathlon-specific factors. Rather, disordered eating behaviors were found to be more often associated with associated competitive athlete factors, particularly in the many leanness sports such as running.

## INTRODUCTION

The prevalence of eating disorders in women in the general population is estimated to be less than 1% for anorexia nervosa (AN), 1-2% for bulimia nervosa (BN) and 3-5% prevalence of partial syndromes.^1^ The current edition of the Diagnostic and Statistical Manual of Mental Disorders (DSM-5) defines AN as a syndrome consisting of restriction of calories leading to significantly low body weight, intense fear of gaining weight and disturbance of the perception of one’s body.^2^ Partial syndromes of eating disorders consist of symptoms that cause clinically significant impairment, but do not meet the full criteria for AN or BN.

In the DSM-5, BN is defined as a syndrome consisting of recurrent episodes of binge eating and recurrent inappropriate compensatory behaviors to prevent weight gain, both occurring at least once a week for two months with self-evaluation influenced by body weight and shape.^2^ The prevalence of eating disorders in males is significantly lower with a male-female ratio for likely under-reported eating disorders estimated between five and 10%.^1^

In athletes, the overall prevalence of eating disorders is higher than the general population,^3^ as well as in female athletes and sports that emphasize leanness such as triathlons.^4,5^ Triathlons are comprised of three sports, swimming, biking and running, in which disordered eating (DE), which encompasses a spectrum of abnormal behavior ranging from mild preoccupation with food and exercise to a diagnosis of AN or BN according to the DSM-5, has been reported.^6–8^ Triathlons are also one of the most rapidly growing sports in the United States.^9^

The governing body of triathlon events in the United States, USA Triathlon (USAT), tracks triathlon participants throughout the country. During the past 10 years, the annual number of purchased USAT memberships has increased from 47,373 in 2003 to 170,033 in 2014.^9^ Based on studies to date, this growing group of triathletes may be at higher risk for DE.^5–8^

For example, in one study using the definition of the “female athlete triad,” 60% of 15 sample women triathletes with ages between 29 and 41 years were in a calorie deficit.^10^ In that study, criteria established prior to re-identification in 2007 by the American College of Sports Medicine were used, which identified a calorie deficit for female athletes as a syndrome characterized by DE, amenorrhea and low bone mineral density. Another study investigating eating behaviors in 188 women triathletes (mean age 35 years) found insufficient caloric intake for energy requirements.^11^

Another study investigating sub-clinical DE characteristics among 393 men (mean age 38 years) and 188 women (mean age 35 years) triathletes showed 58% of the women and 47% of the men reported being dissatisfied with how they perceived their weight.^12^ There may be factors that are unique to triathlon that may contribute to the risk for DE along with factors that can be found in several sports.

Triathlons are generally divided into male and female divisions and have qualifying event criteria which enable amateur athletes to also qualify for national and world championship events. This categorical system of triathlon eligibility may result in increased competitiveness as only a small percentage of triathletes can win a podium placement at championship events. This could put increased pressure on triathletes to perform and thus potentially increase risk for DE.

Triathletes are training more and racing longer distances more frequently than in the past.^9^ Distance race categories include Sprint, Olympic, Half and Full (see Table 1). Although the sprint distances are the most popular, triathletes’ desire to participate in the longest distances appears to increase with experience.^13^ The increased amounts of training typically required for athletes to finish longer triathlons may be associated with higher risk for DE.^12^

**Table 1:** Triathlon Distance Categories

**Race distance**	**Swim length**	**Bike length**	**Run length**
Sprint ^*^	750 m	20 km	5 km
**Olympic**	1500 m	40 km	10 km
**Half Distance**	1.93 km	90.12 km	21 km
**Full Distance**	3.86 km	180.24 km	42.16 km

* Sprint distance triathlons are not always the standard distance.

Nearly one-third (29%) of triathletes utilize some form of structured coaching.^12^ Previous studies have shown that coaches’ possible weight loss recommendations can have a significant influence on athletes.^14^ Additionally, research has shown the opinions of those involved with athletes, including parents, trainers and coaches, can significantly influence an athlete’s opinion of their weight and have an impact on their risk for DE.^15^

Currently, there is little available data concerning DE in triathletes. The purpose of this cross-sectional survey study was to identify the prevalence of DE attitudes and behaviors among a national convenience sample of triathletes. A secondary purpose was to identify “triathlon-specific factors” and “competitive athlete-factors” potentially associated with DE behaviors. (These factor groups will be defined in the Methods section).

Although the vast majority of triathletes are amateur triathletes, the authors hypothesized that certain factors unique to triathlon and competitive athlete factors would be associated with increased rates of DE.

## METHODS

Before data collection, the study had been approved by the Michigan State University Human Research Protection Program. Informed consent language was located on the first page of the survey listing contact information of the researchers and the overall purpose of the study. The first page also outlined that respondents needed to be at least 18 years of age and a current active triathlete in the United States. By completing any or all parts of the survey, participants were interpreted as providing their informed consent. To encourage accurate self-reporting, responses were anonymous.

The online survey link was circulated by emailing an unspecified number of triathlon teams and elite/professional triathletes. Additionally, the survey link was spread on social media, including Facebook and Twitter. Data were exported from Google Form Survey ^16^ software into IBM SPSS Statistics Edition 23 ^17^ for statistical analysis by author Alexander Hayek. The respondent sample was divided into male and female participant subgroups and each set of data was analyzed separately. A chi-square test of independence was performed for each of the triathlon-specific and competitive athlete factors with the Eating Attitudes Test (EAT-26) ^18^ scores treated as a continuous outcome.

The EAT-26 is a standardized self-report measure comprised of three subscales: Dieting, Bulimia, and Food Preoccupation.^19^ The response scale for the EAT-26 items utilizes a six-point Likert Scale: “Never,” “Rarely,” “Sometimes,” “Often,” “Usually” and “Always.” A score of 20 or greater indicates risk for DE.^18^ The total possible score range for the EAT-26 was 0 through 76. For this study, triathletes with a score of 20 or greater were considered to possess some form of DE. The authors’ hypothesized relationships were considered significant if their coefficient Alpha p-value was lower than 0.05.

The triathlon-specific factors that were evaluated included:

- Race Category
- Age Group
- Collegiate, or Elite/Professional status
- Overall Triathlon Finish Category (i.e., top, middle or bottom)
- Championship Qualification Category
- Selected Race Distance
- Years of Triathlon Experience
- Number of Triathlons Per Year
- Sponsorship

The competitive athlete-factors factors that were assessed included:

- Number of sports-related injuries during the past season
- Degree of worry about weight gain during the off season
- Whether or not an athlete was happy with his or her current weight
- Desired amount of weight loss to improve performance
- Specific people who have told the respondent to lose weight (e.g., coach, family member, other triathlete)
- Specific methods of weight loss (e.g., specific dietary restrictions, increase in training volume, increase in training intensity).

The statistical significance of relationships between EAT-26 scores (with a positive EAT-26 score indicating risk of DE) and the triathlon-specific and competitive athlete factors across the vast majority of sports were examined.

The software package GPower 3.0 ^20^ was used to conduct a post-hoc power analysis. The sample size of N=1033, at an alpha level of p < 0.05, and recommended effect sizes parameters of small (w=.1), medium (w=.3), and large (w=.5) were used for the statistical power analyses.^21^ The post-hoc analyses revealed the statistical power for this study was .90 for detecting a small 0.1 effect, and the overall power exceeded .99, much more than the generally observed 1- β minimal level of adequate statistical power (i.e., power of 0.80) to detect meaningful sample subgroup differences.

## RESULTS

A total of 1,033 surveys were completed online and included in the study analyses with largely complete data. However, it is unknown how many surveys may have been initially started but never completed. There were no exclusion criteria observed.

Responding participants included 1,033 triathletes (545 (52.8%) women and 488 (47.2%) men). In terms of 928 respondents’ race age group, there were 435 (46.8%) men, and 493 (53.1%) women. There were 107 (10.3% of total sample) race collegiate (54 men, 53 women) and 70 (6.8%) race elite/professional (34 men, 36 women). There was some reported overlap in these findings between triathlon racing categories because collegiate athletes can also race by age group and elite/professional athletes can race in some collegiate and age group triathlons. This and other socio-demographic information concerning the sample is depicted in Table 2.

**Table 2:** Socio-demographic Sample Characteristics

	**Age group**		**Collegiate**		**Elite/professional**	
	Male	Female	Male	Female	Male	Female
Total,^a^ N (%)	453 (89.1)	493 (90.5)	81 (11)	53 (9.7)	52 (6.9)	36 (6.6)
Mean age,^b^ (years)	41.3±11.3	39.3±11.2	23.8±3.3	22.5±3.3	30.5±5	30.5±7.7
Mean BMI, (kg/m^2^)	24.5±3.2	22.9±3.9	23.1±2.8	21.9±2.6	22.6±2.7	20.8±1.8
Concern/diagnosis ED, N (%)	52 (12)	174 (35.3)	4 (7.4)	24 (45.3)	7 (20.6)	23 (63.9)
Received treatment, N (%)	4 (0.9)	64 (13)	0 (0)	10 (18.9)	3 (8.8)	12 (33.3)
Positive EAT-26,^19^ N (%)	23 (5.3)	95 (19.3)	1 (1.9)	10 (18.9)	1 (2.9)	9 (25)

There is overlap between categories as Collegiate triathletes can race Age Group and Elite/Professional License triathletes can sometimes race Age Group Mean age was calculated using mean age from participant’s age group, which is 18-19, then 5 years intervals (20-24, 25-29, etc.) until the 70+ age group, for which 70 was used.

The authors found that 129 (12.5%) of respondents scored positive on the EAT-26 and 284 (27.5%) were concerned they have an eating disorder or had been diagnosed with an eating disorder. Rates of DE were higher among women (p < 0.001) and triathletes in the 18-19 age group (p <0.001).  Although the rate was highest in the 18-19 age group, it was also imperative to assess DE among other age groups as only 10 (7.8%) of the 129 positive EAT-26 scores belonged to athletes in the 18-19 age group.  There was no significant relationship found between positive EAT-26 scores and a total of 12 former sports (p values ranging from p = 0.80 to 0.06).

The authors had expected a relationship between triathletes who had previously competed in sports that emphasized leanness, such as running, which has been shown in previous research.^22,23^ There was no significant relationship between positive EAT-26 scores and the category “one to two injuries” during the previous season (p = 0.300). There was a significant relationship for the category “three or more injuries or season ended early due to injury” (p < 0.001). With regards to weight loss, there was no significant relationship found between positive EAT-26 scores and the categories “0.45 to 2.3 kg” (p = 0.310), “2.3 to 4.5 kg” (p = 0.190), and “4.5 to 6.8 kg” (p = 0.180). There was a significant relationship for the category “6.8+ kg” (p = 0.002). For methods of weight loss, there was no significant relationship for the category “Increase training hours of intensity” (p = 0.06).

The percent of male and female triathletes for each BMI category (underweight <18.5, normal weight 18.5-24.9, overweight 25-29.9 and obese 30+) were calculated for each of the weight-related factors. These response categories included “unhappy with current race weight,” “told to lose weight by someone who is not a physician” and “told to lose weight by a physician” (Table 3). Multiple participants answered “doctor” or “physician” for the “other” choice on the item “Have you ever been told to lose weight?” survey question. Thus, a new category, “Physician,” was created for project analysis. There was no significant relationship between being told to lose weight by a physician and a positive EAT-26 score, although all other answer choices were significant for women triathletes, including “Coach,” “Another Triathlete,” and “Family Member” (Table 4).

There was also a significant relationship between triathletes who have been diagnosed with an eating disorder or are concerned they have an eating disorder and a positive EAT-26 score (p < 0.001). The authors believe that it is important for readers to consider that 44 (5.7%) sample triathletes who answered “No” to this question had a positive EAT-26 score. This underscores the authors’ assertion that DE or related symptoms/behaviors were often underreported in the sample.

The majority of triathlon-specific factors did not have a significant relationship with a positive EAT-26 score whereas several of the competitive athlete factors did demonstrate a significant relationship with a positive EAT-26 score (Table 4). Of the triathlon-specific factors, only “Olympic” in the category “Race Distance” was found to have a significant relationship with a positive EAT-26 score (p = 0.04). However, the authors believe that this p-value may have been artificially inflated due to the percentage of participants with positive EAT-26 scores in the Olympic category who also reported concerned they have DE or had been diagnosed with an eating disorder (62.5%, of eight respondents) was much higher than the percentage of all study respondents (27.5%).

## DISCUSSION

The results of our study indicated that triathlon-specific factors in this sample were not significantly associated with DE, although competitive athlete factors were associated with DE. Although previous studies have concluded that triathletes may be at higher risks for the onset and maintenance of DE,^7,11,12^ these results suggest that triathlon by itself is not associated with DE. In this study, 129 (12.5%) participants were considered to have DE and 284 (27.5%) were concerned they have an eating disorder or had been diagnosed with an eating disorder.

It should be noted that 964 (93.3%) sample respondents were amateur triathletes and only 87 (9.0%) age group triathletes reported having a precise training plan similar to elite/professional triathletes.^13^ Although age group triathletes were not competing in the elite/professional category, these athletes may be at increased risk for DE due in part to the commonly shared competitive factors associated with DE.

The selected competitive athlete factors included in this design were intended to evaluate the importance of leanness in relation to performance. Leanness has been linked to improved performance in all three disciplines that comprise triathlon. Due to the uniqueness of this combination of disciplines, triathletes may have increased body size dissatisfaction.^24–26^ For example, a study of 487 girl and 468 boy swimmers aged between 9 to 18 found that 75 (15.4%) of the girls and 17 (3.6%) of the boys reported engaging in pathogenic weight control behaviors.^15^

Another study of 61 competitive men cyclists (Mean age 31.6 years) found 12 (19.6%) had DE.^26^ Finally, a study of 181 elite women runners with mean age 29 years found 29 (16%) respondents had DE and 36 (20%) had eating disorders not otherwise specified (DSM-5).^27^ In our study, there was a significant relationship between women former runners and a positive EAT-26 score (p = 0.04).

In our study sample, the majority 566 (54.8%) answered that they are in some way not happy with their current race weight. The majority of both men and women participants who were unhappy with their weight were, however, normal weight or underweight (Table 3). This body dissatisfaction and desire to lose 6.8 kg or more was significantly associated with DE in women triathletes (p = 0.001) and may be due to drive for leanness as triathletes attempt to achieve lower racing weights or ideal body types.

**Table 3:** BMI Frequencies for Weight-Related Questions

	**Male**			
	Underweight	Normal Weight	Overweight	Obese
	<18.5	18.5-24.9	25-29.9	30.0+
Unhappy with weight, N (%^a^)	2 (0.8)	203 (81.9)	35 (14.1)	8 (3.2)
Told to lose weight,^b^ N (%^c^)	0 (0)	50 (42.4)	54 (45.8)	14 (11.9)
Told to lose weight by physician, N (%^d^)	0 (0)	5 (17.2)	15 (51.7)	9 (31)
	**Female**			
	Underweight	Normal Weight	Overweight	Obese
	<18.5	18.5-24.9	25-29.9	30.0+
Unhappy with weight, N (%^a^)	8 (2.5)	226 (71.1)	62 (19.5)	22 (6.9)
Told to lose weight,^b^ N (%^c^)	5 (3.3)	111 (68.9)	17 (44.7)	10 (26.3)
Told to lose weight by physician, N (%^d^)	0 (0)	11 (2.6)	17 (24.3)	10 (43.5)

Percent of those unhappy with current weight Told to lose weight by coach, another triathlete or family member. Percent of those told to lose weight by someone who is not a physician Percent of those told to lose weight by a physician

Additionally, specific methods utilized to lose weight, such as low carbohydrate, low sugar and low fat diets, were associated with DE. Of triathletes with a positive EAT-26 score, 98 (93.3%) of female triathletes and 98 (95.8%) of male triathletes (23 of 24) followed a specific diet (Table 4). The formulation of these diets may represent restrictive eating tendencies and emphasize a good food/bad food structure, which is a known to eventually lead to eating disorders.^24,28^

**Table 4:** Competitive Athlete Factors and Association with EAT-26 ^19^ Score of 20 or Greater

	**Male**		**Female**	
	N (% ^a^)	p	N (% ^b^)	p
Told to lose weight by				
Coach	3 (12.5)	0.65	20 (29.4)	**0.02**
Another triathlete	4 (16.7)	0.25	16 (48.5)	**<0.001**
Family member	2 (8.3)	0.54	27 (31.8)	**0.001**
Injuries during previous season				
3 or more injuries or season ended due to injury	5 (20.8)	**0.02**	25 (45.5)	**<0.001**
Worry during offseason or when cannot train				
Little to few times per week	6 (25)	**0.001^c^**	14 (4.7)	**<0.001^c^**
Daily or constantly	17 (70.8)	**<0.001**	91 (43.5)	**<0.001**
Unhappy with weight	14 (58.3)	0.30	88 (27.5)	**<0.001**
Amount of weight loss desired				
6.8+ kg	3 (12.5)	0.51	20	**0.002**
Method of weight loss				
Specific diet ^d^	23 (95.8)	**<0.001**	98 (26.3)	**<0.001**

Percent reported is out of EAT-26 ^19^ scores of 20 or greater (N=24) Percent reported is out of EAT-26 ^19^ scores of 20 or greater (N=105) Significant that Little or Few associated with negative EAT-26 (score <20) Combination of counting calories, restricting calories, low carbohydrate, low fat, low sugar, high protein and diet pills

DE behaviors can lead to poor nutritional status which may result in increased injuries.^29^ In this sample, triathletes with three or more injuries during the past season were more likely to have positive EAT-26 scores. Additionally, triathletes who have suffered from an injury that ended their prior season prematurely were more likely to be at risk for DE. Therefore, injury frequency during the past season should be considered by sports medicine professionals when evaluating triathlete patients for DE. Reported concerns about gaining weight daily or constantly during the off season or when the triathlete is injured and cannot train was also associated with DE. However, worry about weight gain a little or a few times per week was significantly associated with negative EAT-26 scores. Thus, worry about weight gain a little or a few times per week may indicate a lower risk for DE.

Another factor associated with DE was whether or not a female triathlete had been told or recommended to lose weight. Whether it was a coach, another triathlete or a family member, women triathletes who had been told to lose weight were more likely to have DE (p = 0.01, p = 0.001, and p <0.01; respectively). Of those recommended to lose weight by someone who was not a physician, 50 (42%) of 118 men and 116 (72%) of 143 women were reportedly underweight or normal weight (two far right columns in Table 3).

On the other hand, of those recommended to lose weight by a physician, 24 (83%) of 29 men and 27 (71%) of 38 women reported themselves as being overweight or obese. This is likely because physicians recommend weight loss when a patient is at risk for health complications associated with obesity, whereas triathletes may receive input from coaches regarding body leanness or composition to improve performance. Previous research has shown coaches have significant influence with athletes and coaching style can increase risk for DE and body dissatisfaction.^30,31^ These study results suggest that other triathletes and family members can also have an impact on an athlete’s perception of weight. This may also be compounded by triathletes wishing to race faster and believing increased body leanness will result in improved performance.

This study had several limitations. Due to the cross-sectional design of the study, there may be an excess in prevalence due to long duration of eating disorder cases. The purpose of the study was clearly stated when the survey was administered, which may have caused triathletes with a personal history of or connection to DE to be more or less inclined to complete the survey due to perceived response non-response biases. In addition, this was a self-report survey and thus the answers given may not be entirely accurate.

Another limitation is that the national triathlete population was not proportionately represented in this sample, as only 36.52% of USAT members in 2013 were women and 545 (52.8%) of sample participants were women. Thus, results may be skewed as eating disorders are more prevalent in females than in males. Additionally, the number of men triathletes with positive EAT-26 scores was relatively small (N=24) compared to the number of women triathletes with positive scores (N=105), thus making analysis of features in men triathletes more difficult to generalize to the entire triathlete population.

## CONCLUSIONS

These overall findings suggest that most triathletes can continue to have healthy attitudes about their weights and shapes even when they are competitively racing longer distances. Additionally, these results suggest that triathletes can compete for long periods of time and race more frequently without increased association with DE.

Although triathlon participation by itself was not associated with an increased rate of DE, many triathletes in this study reported competitive athlete factors that were associated with increased rates of DE. Competitive athlete factors are relatively well known to occur in elite runners,^27^ even though responses from this study sample suggests that these factors can occur in all levels of triathlon and may be associated with DE.

Professionals who work closely with athletes on a regular basis (e.g., sports medicine physicians, osteopathic manipulative medicine practitioners and athletic trainers) may have an opportunity to prevent progression from DE to eating disorders as the first professionals to see triathletes with frequent injuries. These professionals may often be in a unique position to screen athletes for DE and facilitate treatment. Future studies to develop and test further methods of identifying and treating triathletes with DE is vital.

### Conflict of Interest

The authors declare no conflict of interest.
